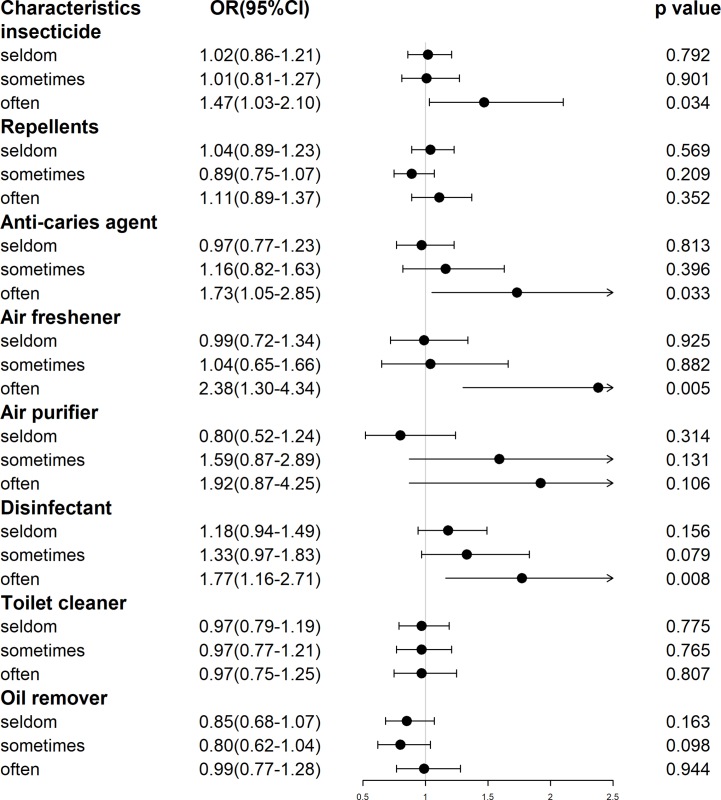# Association of household chemicals use with cognitive function among Chinese older adults

**DOI:** 10.1016/j.heliyon.2024.e37765

**Published:** 2024-09-13

**Authors:** Yanrong Wang, Yongbin Zhu, Yueping Wu, Liping Shi, Yue Yang, Xiaojuan Liu, Jiangping Li

**Affiliations:** a Department of Epidemiology and Health Statistics, School of Public Health, Ningxia Medical University, Yinchuan, Ningxia Hui Autonomous Region, 750004, China; b Key Laboratory of Environmental Factors and Chronic Disease Control, Ningxia Medical University, Yinchuan, Ningxia Hui Autonomous Region, 750004, China

**Keywords:** Household chemical use, Cognitive function, Chinese older adults, Binary regression, Linear regression, Interaction

## Abstract

**Background:**

The paucity of empirical evidence supporting a correlation between the utilization of household chemicals and cognitive decline in Chinese older adults.

**Methods:**

The data utilized for this study originated from the Chinese Longitudinal Healthy Longevity Survey (CLHLS 2018). Using regression models to investigate the relationship between exposure to household chemicals and cognitive decline, and evaluate the impact of different fields on cognitive function.

**Results:**

The use of household chemicals was associated with a decline in cognitive function (anti-caries agent, OR = 1.68, P = 0.040; air freshener, OR = 2.48, P = 0.002; disinfectant, OR = 1.40, P = 0.033). The more frequent the use of household chemicals, the worse the cognitive function (Model1: OR = 2.54, P = 0.024; Model2: OR = 3.23, P = 0.006; Model3: OR = 3.59, P = 0.003).

**Conclusion:**

The study has uncovered a correlation between the utilization of household chemicals and cognitive decline in individuals aged 65 years and over in China.

## Highlights

• The use of household chemicals by elderly people can lead to cognitive decline.
• Combined exposure to the household chemicals products is more important than single product.
• Household chemicals can affect cognitive decline in different dimensions.

## 1 Introduction

Global demographic changes have led to population aging, a significant challenge faced by most countries, including China. By the end of 2019, China had 176 million people over 65 years of age, accounting for 12.6 % of the total population, thus becoming one of the countries with the fastest-growing aging populations [1]. Aging is the greatest risk factor for late-onset Alzheimer's disease (LOAD) [2]. According to the World Alzheimer's Disease Report, more than 46 million people worldwide suffered from dementia in 2015, with this number expected to increase to 75 million by 2030 and 131.5 million by 2050 [3]. Subjective (mild) cognitive decline can be a precursor and distinguishing feature of Alzheimer's disease (AD) and other dementias [4], severely affecting the health-related quality of life of the elderly and placing a significant burden on families and healthcare systems [5]. The global cost of dementia was estimated at $818 billion in 2015, a 35 % increase compared to 2010, with a projected increase to over $1 trillion by 2018 [6]. Focusing on the cognitive health of older adults and supporting successful aging is clinically relevant and socially significant.

The harmful effects of environmental pollutants and fine particulate air pollution on the health of the elderly cannot be ignored [[7], [8], [9]]. There is a correlation between air pollution exposure and poor brain health (clinical dementia, neuroimaging-related, or cognitive impairment) [10]. With the increasing urbanization of the world, people spend more than 90 % of their time indoors [11,12], and the impact of indoor air quality (IAQ) on health status cannot be ignored [13]. Furthermore, the use of cleaning products and disinfectants for infection control and hygiene purposes has increased in recent decades, especially in hospitals and households, resulting in increased indoor air pollution. In France, more than 70 % of women reported cleaning their houses at least once a week [14]. Simultaneously, cleaning products have a specific purpose (detoxifier, sanitizer), resulting in the potential for multiple product usage and mixed health effects [15]. While cognitive function research in older adults has focused on developed countries with relatively low air pollution exposure [[16], [17], [18]], the direct correlation between home cleaning products and cognitive function has not been established.

Therefore, the objective of our study was to investigate the correlation between household chemical use and cognitive function. To achieve this goal, we analyzed the 2018 Chinese Longitudinal Healthy Longevity Survey, a comprehensive survey that encompassed a large sample size and a vast amount of data. We examined the relationship between eight major household chemicals, including insecticides, repellents, anti-caries agents, air fresheners, air purifiers, disinfectants, toilet cleaners, and oil removers, and the risk of cognitive impairment in the Chinese elderly population (aged ≥65 years), and constructed a comprehensive household chemicals score to elucidate the dose-response relationship between the frequency and quantity of household chemicals employed and the susceptibility to cognitive dysfunction.

## 2 Methods

### 2.1 Data source and sample

The data utilized for this study originated from the Chinese Longitudinal Healthy Longevity Survey (CLHLS 2018). CLHLS, an extensive follow-up survey of the Chinese elderly population, was coordinated by the Center for Healthy Aging and Development Studies (CHADS) of Peking University and initiated in 1998. The survey subjects included individuals aged 65 and above residing in 23 Chinese provinces. Furthermore, additional information pertaining to the CLHLS survey has been meticulously detailed in previous studies [19]. According to the research objectives, we meticulously excluded individuals under the age of 65 and those with missing data on the study factors. Ultimately, the sample consisted of 10,387 individuals aged 65 and above, who had no somatoform disorders and provided complete data relevant to the research questions.

### 2.2 Cognitive function

Cognitive function was assessed using the Chinese version of the Mini-Mental State Examination (MMSE), which serves as a screening tool for the assessment of cognitive function and the degree of deficit [20,21]. The MMSE is also commonly used as a screening tool for dementia, displaying high specificity (usually exceeding 0.80) and moderate reliability (Pearson correlation coefficient of 24-h test-retest usually surpassing 0.85) according to a meta-analysis conducted in 2013 [22]. We utilized a customized version of the MMSE, tailored to the specific cultural and socioeconomic conditions prevalent in China [23]. The MMSE assesses cognitive function across five domains, namely orientation, registration, attention and calculation, recall, and language [24]. Each item was scored as zero (incorrect or unable to answer) or one (correct), with a total score ranging from 0 to 30 [25]. Scores reflecting higher levels of cognitive function were associated with higher values [26]. Given that more than half of the participants were illiterate, a relatively low cutoff score of 18 was employed to classify individuals with poor cognitive function, and scores ≥18 were deemed indicative of normal cognitive function [1,26,27]. Additionally, when further evaluating the relationship between the use of household chemicals and different dimensions of cognitive function, the scores of the five dimensions are considered as continuous variables.

### 2.3 Household chemical usage exposure

The CLHLS questionnaire categorizes household chemicals into eight distinct categories: insecticides, repellents, anti-caries agents, air fresheners, air purifiers, disinfectants, toilet cleaners, and oil removers. The use of these chemicals is further elucidated through the question: Do you utilize the aforementioned chemicals in your home? Respondents were prompted to select one of four response options, including: rarely or never, seldom, sometimes, and often. We will use binary regression to evaluate the impact of different usage frequencies of meaningful household chemicals on cognitive function. Based on the results, it can be inferred that these three meaningful household chemicals are anti-caries agents, air fresheners, and disinfectants. Considering these three as total effect variables, we define the frequency of use of each chemical from seldom to often as the high-frequency use group. Due to its combination frequency scoring range of 3–12 points, the group with a score of 3–9 points is defined as the low-frequency group, and the group with a score of 9–12 points is defined as the high-frequency group.

### 2.4 Covariates

**Fig. 1:**
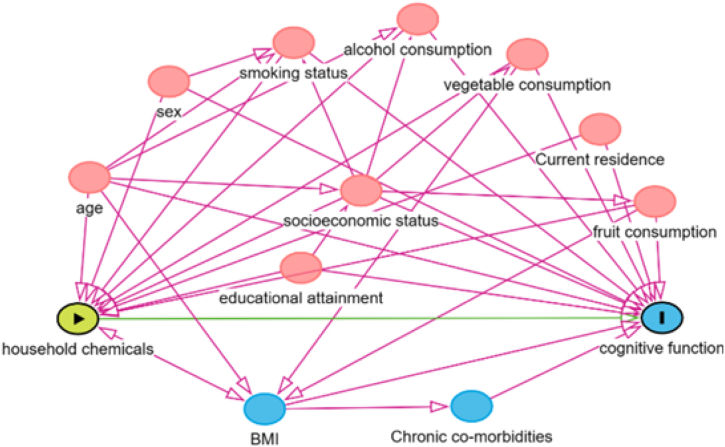
A directed acyclic graph (DAG) represents the associations between covariates and primary exposure and outcome. The pink circles represent the ancestors of the exposure and outcome (ie, confounders), while the blue circles represent the ancestors of the outcome (ie, causal determinants of the outcome). Green lines represent causal paths, and pink lines represent biasing paths. The minimally sufficient adjustment set represents covariates that minimize confounding bias when estimating the association between the exposure and the outcome, as determined by utilizing the DAGitty software. The final minimally sufficient adjustment set comprised age, sex, current residence, body mass index, educational attainment, socioeconomic status, smoking status, alcohol consumption, fruit consumption, and vegetable consumption.

Potential confounders and effect modifiers were identified from the existing literature and incorporated into a directed acyclic graph (DAG) that served as a guiding framework for the modeling strategy (Fig. 1) [[28], [29], [30], [31], [32], [33], [34]]. These covariates encompassed demographic characteristics (age, gender, and place of residence), socioeconomic characteristics (level of education, economic status), behavioral habits (smoking, alcohol consumption, and vegetable and fruit intake), and health status (body mass index, BMI). The place of residence was classified into three categories: city, town, and village. Educational attainment was categorized into four distinct levels corresponding to the duration of schooling: level zero (illiteracy), level one (primary school), level two (middle school), and level three (college degree or higher). Economic status was categorized into three distinct categories: level one (good), level two (general), and level three (bad). Tobacco and alcohol consumption were dichotomized as either “ONE” (yes) or “ZERO” (no). Vegetable and fruit intake were also categorized into four distinct levels: level one (almost daily), level two (except during the winter), level three (occasional), and level four (rare or never).

### 2.5 Statistics analysis

The data were subjected to rigorous statistical analysis using R4.3.1. All tests were conducted in a two-tailed manner, and a p-value <0.05 was deemed statistically significant. Continuous variables were expressed as means ± standard deviations or medians (interquartile ranges, IQRs), while counts and percentages were utilized to represent categorical variables. T-tests and chi-square tests were utilized to compare the baseline characteristics between two groups: patients with cognitive impairment and those without cognitive impairment.

Binary logistic regression was used to explore the association between categorical cognitive function and household chemical use, while linear regression analysis was employed to investigate the relationship between continuous cognitive dimensions and household chemical use. Model 1 adjusted for demographic characteristics; Model 2 adjusted for socio-economic characteristics; Model 3 further controlled for health-related behaviors.

Lastly, to assess the robustness of the findings, multiple sensitivity analyses were conducted. Firstly, when evaluating data quality, the CLHLS survey project team suggested that there may be biases in the information reported by researchers on elderly individuals aged 105 and above [35]. Consequently, we excluded research subjects aged >105 years and performed logistic regression analysis and linear regression analysis. Secondly, we grouped the genders and conducted logistic and linear regression analyses to explore the differences in the frequency of household chemicals use between genders. Thirdly, we reported the E value utilizing the methods proposed by VanderWeele and Ding [36,37]. A directed acyclic graph (Fig. 1) was utilized to identify the most appropriate set of adjustments.

## 3 Results

### 3.1 Sample characteristics

Table 1 depicts the demographic characteristics of the study population. Table 2 illustrates the utilization patterns of eight household chemicals. In stark contrast, the top three most commonly employed household chemicals are insecticides, repellents, and toilet cleaners, while the least frequently utilized items encompass air purifiers and air fresheners. Results of the univariate analysis unveiled a notable correlation between cognitive function and anti-caries agents, air purifiers, toilet cleaners, and oil removal. Furthermore, a one-way analysis was conducted to assess the total scores obtained by summing up the scores attributed to the eight household chemicals based on their respective categorizations, which also demonstrated a significant correlation with cognitive function.

**Table 1:** Description of demographic variables in the study sample (n = 10,387).

Subgroups	Total sample	Cognitive		t/χ^2^	p
		+	–		
**Total sample (n(%))**	10,387	1595(15.4)	8792(84.6)		
**Age (year, mean ± SD)**	84.3 ± 11.6	96.5 ± 7.3	82.1 ± 10.9	−50.802	<0.001
**Sex (n(%))**				194.01	<0.001
male	4653(44.8)	460(28.8)	4193(47.7)		
female	5734(55.2)	1135(71.2)	4599(52.3)		
**Current residence(n(%))**				45.05	<0.001
City	2503(24.1)	279(17.5)	2224(25.3)		
town	3499 (33.7)	589(36.9)	2910(33.1)		
Rural	4385(42.2)	727(45.6)	3658(41.6)		
**Educational attainment(n(%))**				814.40	<0.001
illiteracy	4918 (47.4)	1275(79.9)	3643(41.4)		
Primary school	3427(33.0)	244(15.3)	3183(36.2)		
Middle school	1665(16.0)	65(4.1)	1600(18.2)		
College degree or above	377(3.6)	11(0.7)	366(4.2)		
**Socioeconomic status(n(%))**				69.63	<0.001
Good	2018(19.4)	213(13.4)	1805(20.5)		
So so	7315(70.4)	1153(72.3)	6162(70.1)		
Bad	1054(10.2)	229(14.4)	825(9.4)		
**Smoking status(n(%))**				94.17	<0.001
No	7214(69.5)	1272(79.8)	5942(67.6)		
Yes	3173(30.6)	323(20.3)	2850(32.4)		
**Alcohol consumption(n(%))**				56.45	<0.001
No	7662(73.8)	1298(81.4)	6364(72.4)		
Yes	2725(26.2)	297(18.6)	2428(27.6)		
**Fruit consumption(n(%))**				131.33	<0.001
Almost everyday	2318(22.3)	247(15.5)	2071(23.6)		
Except winter	2433(23.4)	330(20.7)	2103(23.9)		
occasionally	3155(30.4)	471(29.5)	2684(30.5)		
Rarely or never	2481(23.9)	547(34.3)	1934(22.0)		
**Vegetable consumption(n(%))**				385.86	<0.001
Almost everyday	6802(65.5)	815(51.1)	5987(68.1)		
Except winter	2486(23.9)	410(25.7)	2076(23.6)		
occasionally	755(7.3)	219(13.7)	536(6.1)		
Rarely or never	344(3.3)	151(9.5)	193(2.2)		
**BMI(n(%))**				393.78	<0.001
<18.5	1709(16.5)	512(32.1)	1197(13.6)		
[18.5–24)	5364(51.6)	793(49.7)	4571(52.0)		
[24–28)	2467(23.8)	224(14.0)	2243(25.5)		
≥28	847(8.2)	66 (4.1)	781(8.9)		

Note: SD, standard deviation; BMI, body mass index; +, Cognitive impairment; -, No cognitive impairment.

**Table 2:** Description of exposure variables in the study sample (n = 10387).

Characteristics	Total sample	Cognitive		t/χ^2^	p
		+	–		
**Insecticide(n(%))**				7.18	0.066
never	6839(65.8)	1094(68.6)	5745(65.3)		
seldom	2100(20.2)	293(18.4)	1807(20.6)		
sometimes	1080(10.4)	150(9.4)	930(10.6)		
often	368(3.5)	58(3.6)	310(3.5)		
**Repellents(n(%))**				5.18	0.159
never	4132(39.8)	674(42.3)	3458(39.3)		
seldom	2676(25.8)	402(25.2)	2274(25.9)		
sometimes	2214(21.3)	319(20.0)	1895(21.6)		
often	1365(13.1)	200(12.5)	1165(13.3)		
**Anti-caries agent(n(%))**				10.44	0.015
never	8729(84.0)	1382(86.7)	7347(83.6)		
seldom	1012(9.7)	126(7.9)	886(10.1)		
sometimes	447(4.3)	57(3.6)	390(4.4)		
often	199(1.9)	30(1.9)	169(1.9)		
**Air freshener(n(%))**				6.79	0.079
never	9515(91.6)	1474(92.4)	8041(91.5)		
seldom	562(5.4)	70(4.4)	492(5.6)		
sometimes	212(2.0)	30(1.9)	182(2.1)		
often	98(0.9)	21(1.3)	77(0.9)		
**Air purifier(n(%))**				10.22	0.017
never	9854(94.9)	1531(96.0)	8323(94.7)		
seldom	344(3.3)	33(2.1)	311(3.5)		
sometimes	130(1.3)	19(1.2)	111(1.3)		
often	59(0.6)	12(0.8)	47(0.5)		
**Disinfectant(n(%))**				4.77	0.189
never	8564(82.5)	1344(84.3)	7220(82.1)		
seldom	1046(10.1)	139(8.7)	907(10.3)		
sometimes	507(4.9)	72(4.5)	435(5.0)		
often	270(2.6)	40(2.5)	230(2.6)		
**Toilet cleaner(n(%))**				34.86	<0.001
never	6699(64.5)	1130(70.9)	5569(63.3)		
seldom	1472(14.2)	195(12.2)	1277(14.5)		
sometimes	1182(11.4)	152(9.5)	1030(11.7)		
often	1034(1.0)	118(7.4)	916(10.4)		
**Oil remover(n(%))**				49.84	<0.001
never	7242(69.7)	1230(77.1)	6012(68.4)		
seldom	1211(11.7)	150(9.4)	1061(12.1)		
sometimes	942(9.1)	102(6.4)	840(9.6)		
often	992(9.6)	113(7.1)	879(10.0)		
**Total score(mean ± SD)**	11.6 ± 3.5	11.1 ± 3.5	11.6 ± 3.5	5.39	<0.001

Note: SD, standard deviation; Total score, Total score for the eight household chemicals combined; +, Cognitive impairment; -, No cognitive impairment.

### 3.2 Relationship between household chemical usage and cognitive function in multifactorial binary regression

**Fig. 2:**
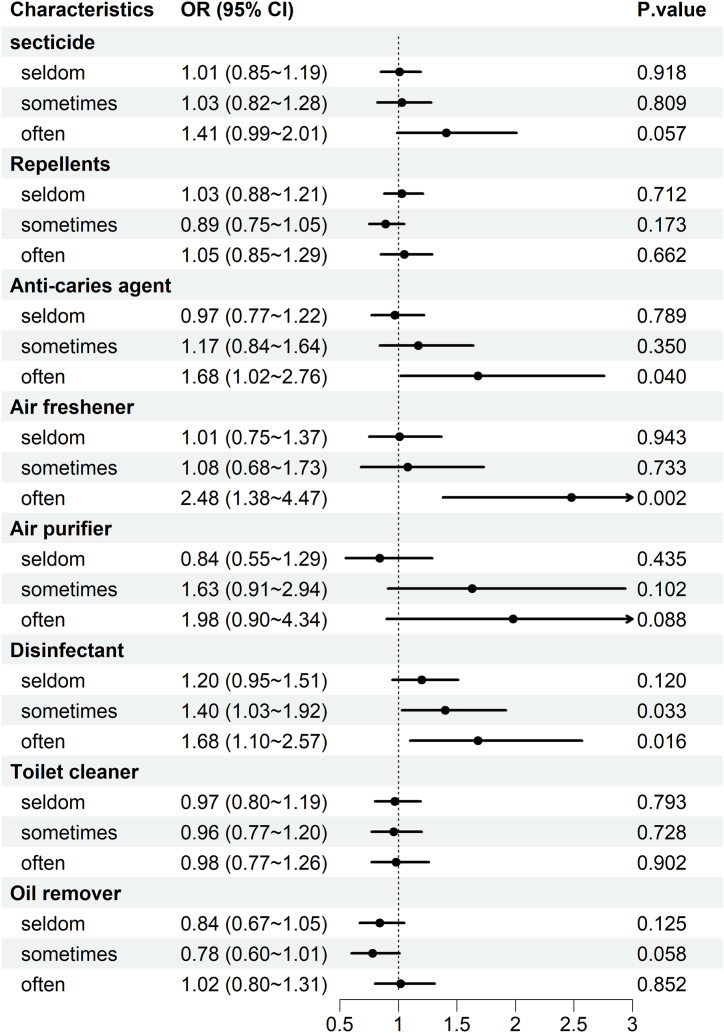
Utilizing a binary regression model to investigate the association between household chemical exposure and cognitive function scores. Adjustment for factors such as age, sex, current residence, body mass index, educational attainment, socioeconomic status, smoking status, alcohol consumption, fruit consumption, and vegetable consumption.

Fig. 2 displays the correlation between household chemical use and cognitive function. After controlling for various covariates such as age, gender, body mass index (BMI), residential location, educational attainment, economic status, smoking habits, alcohol consumption, and dietary intake of vegetables and fruits. Our analysis revealed a significant correlation between three types of household chemicals and cognitive function at varying usage levels. Specifically, the use of anti-caries agents (classified as frequent or non-use) exhibited a correlation of 1.68 (95 % CI = 1.02–2.76, P = 0.040), air fresheners (classified as frequent or non-use) exhibited a correlation of 2.48 (95 % CI = 1.38–4.47, P = 0.002), and disinfectants (classified as occasional or non-use) exhibited a correlation of 1.40 (95 % CI = 1.03–1.92, P = 0.033), as well as a correlation of 1.68 (95 % CI = 1.10–2.57, P = 0.016) for frequent use. From this analysis, it is evident that a negative monotonic correlation exists between the use of household chemicals and cognitive function, suggesting that an increase in chemical exposure is associated with a decrease in cognitive performance.

To further analyze the relationship between anti-caries agents, air fresheners, and disinfectants and cognitive function, we divided the total effects of these three chemicals into two categories (low-frequency and high-frequency) based on their frequency of use, and evaluated them using three logistic regression models. The subsequent results indicate that compared to low-frequency use of household chemicals, high-frequency use of these chemicals is more stronger associated with cognitive decline (Model 1: OR = 2.54, 95 % CI = 1.13–5.70, P = 0.024; Model 2: OR = 3.23, 95 % CI = 1.40–7.48, P = 0.006; Model 3: OR = 3.59, 95 % CI = 1.54–8.38, P = 0.003) (Table 3). The predictive validity of the model is provided in the supplementary material (Supplementary material).

**Table 3:** A binary regression test was conducted to investigate the correlations between the frequency of household chemicals use and the cognitive function score.

Characteristics	Model 1			Model 2			Model 3		
	OR	95%CI	*P*	OR	95%CI	*P*	OR	95%CI	*P*
**Total effect**									
**Low-frequency**	Ref.			Ref.			Ref.		
**High-frequency**	2.54	1.13–5.70	0.024	3.23	1.40–7.48	0.006	3.59	1.54–8.38	0.003

Note: Model 1 adjusts for age, sex, current residence, and body mass index; Model 2 adjusts for educational attainment and socioeconomic status; Model 3 adjusts for smoking status, alcohol consumption, fruit consumption, and vegetable consumption.

### 3.3 Linear regression between household chemical usage and cognitive function in different dimensions

Based on the previous findings, it was observed that the relationship between chemical exposure and cognitive function exhibited a negative correlation, indicating that the use of chemicals poses a potential risk factor for cognitive function. Cognitive function was evaluated across five domains, including orientation, registration, attention and calculation, recall, and language [24], in order to discern the specific domain responsible for cognitive decline. To this end, the influence of chemicals was analyzed separately within each of these five domains to investigate their respective effects on cognitive function. As presented in Table 4, the use of multiple chemicals exerted a significant impact on registration (β coefficient (95 % CI) = −0.06(−0.11,-0.01), P < 0.05; β coefficient (95 % CI) = −0.21(−0.38, −0.03), P < 0.05; β coefficient (95 % CI) = −0.24(−0.47, −0.01), P < 0.05; β coefficient (95 % CI) = −0.07(−0.13,-0.01), P < 0.05) and recall (β coefficient (95 % CI) = -0.12(−0.18,-0.05), P < 0.01; β coefficient (95 % CI) = -0.18(−0.34, −0.03), P < 0.05; β coefficient (95 % CI) = -0.26(−0.48, −0.05), P < 0.05) domains. On the other hand, the influence on the orientation (β coefficient (95 % CI) = -0.64(−1.13, −0.15), P < 0.05) and language (β coefficient (95 % CI) = -0.47(−0.83, −0.11), P < 0.05) domains was relatively minimal.

**Table 4:** Linear correlation between cognitive function and household chemicals usage in different domains.

Characteristics	Orientation	Registration	Calculation	Recall	Language
I**nsecticide**	−0.19(-0.45, 0.07)	−0.09(-0.18, 0.01)	−0.023(-0.42, −0.05)∗∗	−0.11(-0.22, 0.01)	−0.06(-0.21, 0.08)
**Repellents**	−0.01(-0.14, 0.14)	−0.06(-0.11, −0.01)∗∗	−0.01(-0.12, 0.09)	−0.12(-0.18, −0.05)∗∗∗	−0.01(-0.09, 0.07)
**Anti-caries agent**	−0.09(-0.44, 0.25)	−0.08(-0.21, 0.04)	−0.02(-0.27, 0.22)	−0.18(-0.34, −0.03)∗∗	−0.13(-0.32, 0.07)
**Air freshener**	−0.64(-1.13, −0.15)∗∗	−0.21(-0.38, −0.03)∗∗	−0.33(-0.68, 0.02)	−0.26(-0.48, −0.05)∗∗	−0.25(-0.53, 0.03)
**Air purifier**	−0.60(-1.23, 0.03)	−0.24(-0.47, −0.01)∗∗	−0.18(-0.63, 0.28)	−0.28(-0.56, 0.01)	−0.47(-0.83, −0.11)∗∗
**Disinfectant**	−0.11(-0.41, 0.19)	−0.08(-0.19, 0.03)	−0.24(-0.46, −0.03)∗∗	−0.10(-0.24, 0.03)	−0.17(-0.34, 0.01)
**Toilet cleaner**	0.08(-0.08, 0.24)	−0.02(-0.08, 0.04)	−0.05(-0.17, 0.06)	−0.06(-0.14, 0.01)	−0.02(-0.11, 0.07)
**Oil remover**	−0.09(-0.26, 0.07)	−0.07(-0.13, −0.01)∗∗	0.03(-0.09, 0.15)	−0.04(-0.11, 0.04)	−0.07(-0.17, 0.02)

Note: numerical representation β coefficient (95 % CI); ∗∗, P < 0.05; ∗∗∗, P < 0.01.

### 3.4 Stratified analysis

**Fig. 3:**
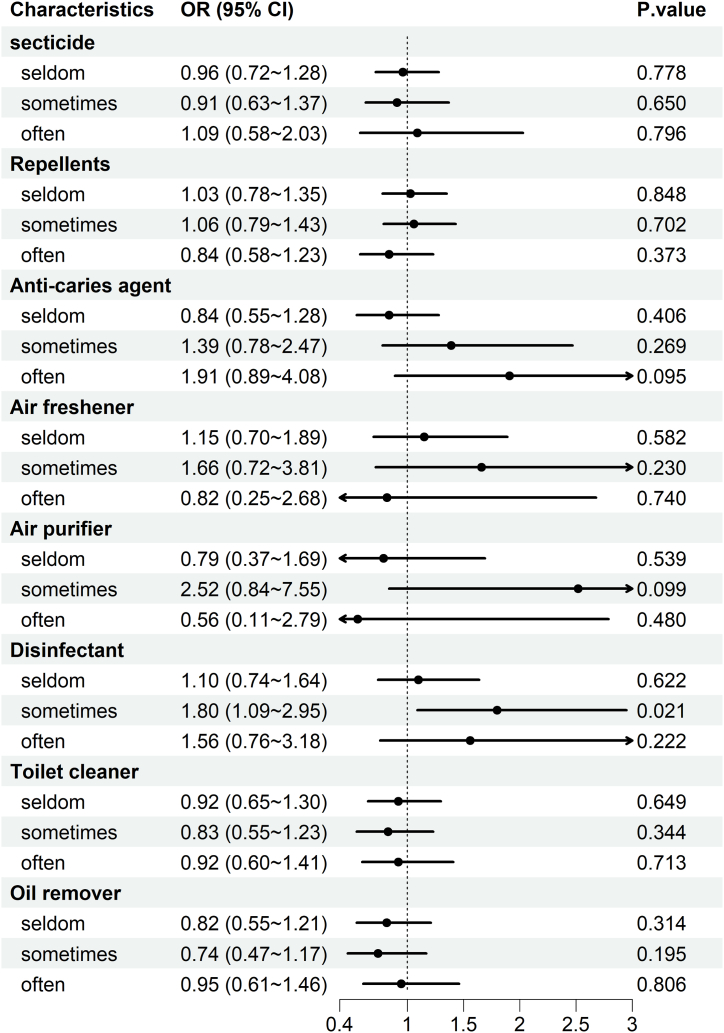
A binary regression model was employed to examine the correlation between household chemicals usage and cognitive function scores in male participants. Adjust for age, sex, current residence, body mass index, educational attainment, socioeconomic status, smoking status, alcohol consumption, fruit consumption, and vegetable consumption.

**Fig. 4:**
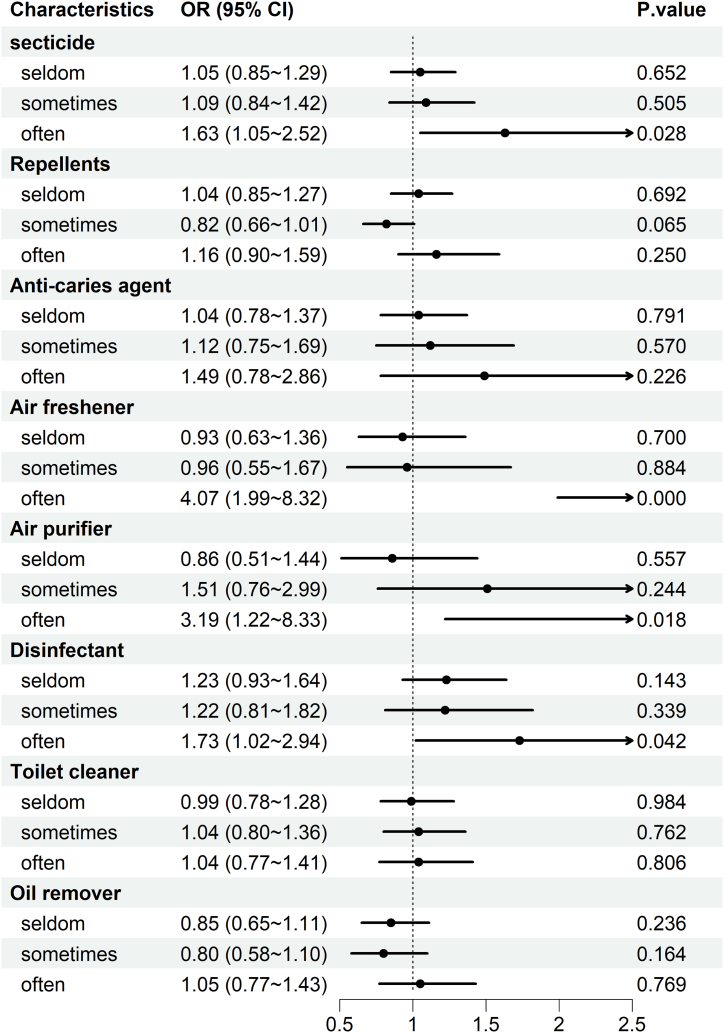
Binary regression model to investigate the correlation between household chemical exposure and cognitive function in women. Adjust for age, sex, current residence, body mass index, educational attainment, socioeconomic status, smoking status, alcohol consumption, fruit consumption, and vegetable consumption.

In this study, stratified analysis was employed to analyze both male and female participants (Fig. 3, Fig. 4), and the results revealed that chemical use among males was not significantly correlated with cognitive function (Model3: OR = 1.46, 95 % CI = 0.72–2.97, P = 0.291). Conversely, there was a significant correlation between female chemical use and cognitive function (Model3: OR = 3.40, 95 % CI = 1.26–9.16, P = 0.015) (Table 5). The predictive validity of the model is provided in the supplementary material (Supplementary material). Among the five dimensions, male showed a negative correlation with chemical use only in the recall dimensions, while female demonstrated negative correlations with attention, calculation, and recall dimensions(Table 6). This suggests that the use of household chemicals is more likely to cause declines in the recall dimensions in males and in the dimensions of attention, calculation, and recall in females.

**Table 5:** Correlation test for household chemicals and cognitive functioning scores by gender using binary regression.

Subgroup	Model 1			Model 2			Model 3		
	OR	95%CI	*P*	OR	95%CI	*P*	OR	95%CI	*P*
**Male total effect**									
**Low- frequency**	Ref.			Ref.			Ref.		
**High-frequency**	1.19	(0.61–2.34)	0.609	1.39	(0.69–2.79)	0.352	1.46	(0.72–2.97)	0.291
**Female total effect**									
**Low- frequency**	Ref.			Ref.			Ref.		
**High-frequency**	2.70	(1.02–7.15)	0.046	3.21	(1.19–8.69)	0.021	3.40	(1.26–9.16)	0.015

Note: Model 1 adjusts for age, current residence, and body mass index; Model 2 adjusts for educational attainment and socioeconomic status; Model 3 adjusts for smoking status, alcohol consumption, fruit consumption, and vegetable consumption.

**Table 6:** A linear relationship between cognitive function and household chemical usage across gender.

Characteristics	Orientation	Registration	Calculation	Recall	Language
M**ale**					
I**nsecticide**	−0.23(-0.56, 0.11)	−0.08(-0.20, 0.04)	−0.09(-0.34, 0.16)	−0.06(-0.22, 0.10)	−0.11(-0.30, 0.08)
**Repellents**	0.08(-0.11, 0.27)	−0.01(-0.07, 0.06)	0.02(-0.12, 0.16)	−0.12(-0.21, −0.02)∗∗	0.03(-0.07, 0.14)
**Anti-caries agent**	−0.27(-0.72, 0.18)	−0.03(-0.19, 0.14)	0.04(-0.29, 0.37)	−0.04(-0.25, 0.18)	−0.17(-0.42, 0.07)
**Air freshener**	−0.23(-0.88, 0.41)	−0.05(-0.29, 0.18)	−0.07(-0.54, 0.41)	−0.16(-0.47, 0.15)	−0.08(-0.44, 0.28)
**Air purifier**	−0.39(-1.21, 0.42)	−0.11(-0.41, 0.19)	0.11(-0.50, 0.71)	−0.09(-0.49, 0.30)	−0.29(-0.75, 0.16)
**Disinfectant**	−0.14(-0.54, 0.26)	−0.04(-0.18, 0.11)	0.01(-0.28, 0.31)	0.01(-0.19, 0.19)	−0.04(-0.26, 0.18)
**Toilet cleaner**	0.01(-0.20, 0.22)	0.02(-0.06, 0.09)	−0.01(-0.16, 0.16)	−0.03(-0.13, 0.08)	0.04(-0.08, 0.16)
**Oil remover**	−0.19(-0.41, 0.03)	−0.06(-0.14, 0.02)	−0.08(-0.24, 0.08)	−0.02(-0.13, 0.08)	−0.02(-0.14, 0.10)
F**emale**					
I**nsecticide**	−0.16(-0.54, 0.22)	−0.10(-0.24, 0.04)	−0.37(-0.64, −0.11)∗∗	−0.16(-0.32, 0.01)	−0.03(-0.25, 0.19)
**Repellents**	−0.08(-0.28, 0.13)	−0.11(-0.19, −0.04)∗∗	−0.06(-0.20, 0.09)	−0.13(-0.21, −0.04)∗∗	−0.05(-0.17, 0.06)
**Anti-caries agent**	0.02(-0.49, 0.53)	−0.14(-0.33, 0.04)	−0.10(-0.47, 0 0.26)	−0.32(-0.54, −0.10)∗∗	−0.11(-0.40, 0.18)
**Air freshener**	−0.95(-1.67, −0.23)∗∗	−0.33(-0.58, −0.07)∗∗	−0.56(-1.07, −0.05)∗∗	−0.35(-0.66, −0.05)∗∗	−0.37(-0.78, 0.04)
**Air purifier**	−0.70(-1.64, 0.24)	−0.33(-0.67, 0.01)	−0.42(-1.08, 0.25)	−0.45(-0.85, −0.05)∗∗	−0.56(-1.10, −0.02)∗∗
**Disinfectant**	−0.07(-0.51, 0.36)	−0.12(-0.27, 0.04)	−0.46(-0.77, −0.15)∗∗	−0.19(-0.38, −0.01)∗∗	−0.26(-0.51, −0.01)∗∗
**Toilet cleaner**	0.14(-0.10, 0.38)	−0.05(-0.14, 0.03)	−0.12(-0.29, 0.05)	−0.11(-0.21, −0.01)∗∗	−0.08(-0.22, 0.05)
**Oil remover**	0.02(-0.23, 0.26)	−0.07(-0.15, 0.02)	0.12(-0.05, 0.29)	−0.05(-0.15, 0.06)	−0.10(-0.24, 0.04)

Note: numerical representation β coefficient (95 % CI); ∗∗, P < 0.05; ∗∗∗, P < 0.01.

### 3.5 Interaction between household chemicals

In multivariate analysis, an OR value of 1.12 indicates a positive interaction effect, suggesting that simultaneous exposure to both anti-caries agents and disinfectants is associated with a higher risk of cognitive decline compared to exposure to either anti-caries agents or disinfectants alone(P < 0.05) (Table 7).

**Table 7:** Analysis of the interactive effects of household chemicals on cognitive decline.

Interaction term	OR (95%CI)	*P*
**Air-freshener by Anti-caries agent**	1.06(0.92–1.22)	0.399
**Air-freshener by Disinfectant**	1.03(0.91–1.16)	0.680
**Anti-caries agent by Disinfectant**	1.12(1.01–1.25)	0.033
**Air-freshener by Anti-caries agent by Disinfectant**	1.02(0.99–1.05)	0.155

Note: Adjust for age, sex, current residence, body mass index, educational attainment, socioeconomic status, smoking status, alcohol consumption, fruit consumption, and vegetable consumption.

### 3.6 Sensitivity analysis

A small number of individuals aged 105 and above were excluded from the study [35]. The results obtained were consistent with those previously observed (Model1: OR = 2.29, 95 % CI = 1.11–4.73, P = 0.025; Model2: OR = 2.63, 95 % CI = 1.25–5.55, P = 0.011; Model3: OR = 2.78, 95 % CI = 1.31–5.91, P = 0.008) (Supplementary material). Simultaneously, the E-value was employed as a supplement to the robust analysis of the study. The E-value is defined as the minimum strength of association, on the risk ratio scale, that an unmeasured confounder would need to have with both the exposure and the outcome to fully explain away a specific exposure-outcome association, conditional on the measured covariates [36,37]. In our results, it was found that high-frequency use of anti-caries agents, air fresheners, and disinfectants increased the risk of cognitive decline by 3.59-fold compared to low-frequency use. The calculated E value was 6.64, indicating that any other unmeasured confounding factors must have an odd ratio to cognitive function of at least 6.64, and this strength of unmeasured confounding is almost non-existent in rigorously designed studies, making this result robust.

## 4 Discussion

This cross-sectional analysis conducted a nationwide representative sampling survey on a total of 10,387 Chinese elderly individuals aged 65 and above to explore the association between the frequency of household chemical use and cognitive function. The research findings confirm the hypothesized relationship, whereby elderly individuals who frequently employ anti-caries agents, air fresheners, or disinfectants exhibit a higher risk of cognitive decline when controlling for relevant confounders. Furthermore, the overall usage burden of various household chemicals also appears to be linked to the risk of cognitive decline, with varying degrees of impact on distinct domains of cognitive function.

Cleaning products encompass a diverse array of ingredients that have the potential to cause airway irritations (bleach, ammonia, solvents, acids) [38] or elicit allergic responses (fragrances such as limonene) [39,40], thereby precipitating or exacerbating asthma [[41], [42], [43]]. Despite being a pulmonary disorder, asthma may have deleterious effects on the central nervous system as the brain tissue exhibits inherent sensitivity to oxygen deprivation. During an acute asthma attack, oxygen saturation can descend to persistently low levels [44], and severe exacerbations can give rise to various degrees of diffuse cerebral hypoxia, potentially resulting in loss of consciousness, cyanosis, hypoxic brain damage, and even fatality [45,46]. Neurological alterations resulting from hypoxia/ischemia in susceptible brain regions with high metabolic demands (e.g., neocortex, hippocampus, basal ganglia) can subsequently give rise to cognitive dysfunction. Recent investigations have revealed that individuals with asthma experience higher rates of cognitive and memory impairment throughout their lifespan compared to healthy populations. A meta-analysis encompassing a collective sample size of over 4148 participants revealed a moderate effect size correlation between asthma and impaired cognitive function [47], with individuals over the age of 55 in a large community sample exhibiting an approximately 78 % increased risk of experiencing mild cognitive impairment [48]. The constituents emitted by air fresheners include benzene, phthalates, and limonene, which can also react with ozone to generate secondary pollutants such as formaldehyde, secondary organic aerosols (SOAs), oxidation products, and ultrafine particles. These secondary pollutants possess the capacity to exert adverse effects on human health, particularly by damaging the central nervous system [49].

In our study, it was evident that the correlation between genders was significantly different, which may be attributed to the fact that women are more extensively involved in household cleaning [14], and employ chemicals with increased frequency, ultimately leading to a conclusion that aligns with our study. With respect to the dimensionality of the phenomenon, gender disparities in cognitive ability have been demonstrated [50], thereby exerting varying effects that merit further exploration through research. Within the familial environment, the utilization of multiple cleaning products to complete all cleaning tasks in succession may have a mixed impact on health [15]. Furthermore, our study also corroborates the likelihood that the combination of certain cleaning agents may engender a mixed effect on health.

One of the limitations of our research lies in its cross-sectional study design, which may impede the accurate illustration of causality and potentially produce spurious correlations. The generalizability of our findings to the relationship between long-term cognitive decline and household chemical use is limited, given the lack of longitudinal data. Furthermore, several potential covariates, either unmeasured (e.g., presence of chronic diseases, level of social activity, marital status) or unknown, may act as confounders in the association between cognitive decline and mortality. Nevertheless, we took into consideration a comprehensive set of common confounders, which resulted in robust findings. Residual confounding remains a potential concern.

On the positive side, this study stands out for its substantial sample size, its representation of participants drawn from randomly selected counties and cities across 22 out of the 31 provinces, autonomous regions, and municipalities in the country, and its widely acknowledged data quality by scholars both domestically and internationally. To our knowledge, this is the first study to examine the correlation between cognitive decline and household chemical exposure. The current investigation encompassed a substantial number of Chinese adults aged 65 years and older, thereby providing a robust evaluation of the relationship between cognitive decline and household chemical exposure in this age group.

Given that the cognitive function in older adults can be amenable to modification [51], the present findings hold significant public health implications. The results manifest the practical significance of utilizing the MMSE or other abbreviated assessment instruments to detect cognitive decline in community settings, particularly among relatively young Chinese older adults and individuals exhibiting normal cognitive function.

For strategies and interventions aimed at reducing the risk of cognitive decline in the elderly population, specific preventive measures may include promoting the use of harmless or low-toxicity household chemicals, enhancing public awareness and education to improve elderly individuals' understanding of the safe use of household chemicals, advocating the use of these chemicals in well-ventilated areas and recommending the use of masks and gloves during application, and advising individuals to leave the area after cleaning and avoid prolonged exposure to minimize contact with household chemicals, thereby achieving preventive effects.

## 5 Conclusion

The utilization of household chemicals has been associated with cognitive decline in Chinese older adults aged 65 and above. To the best of our knowledge, this is the first study to assess the correlation between the usage of household chemicals and cognitive function in Chinese older adults. This study substantiates the need for vigilant monitoring of cognitive alterations in older adults.

## Funding statement

This work was supported by the Key R&D projects in Ningxia (2021BEB04022), 10.13039/501100004772Ningxia Natural Science Foundation (2022AAC03174) and 10.13039/501100001809National Natural Science Foundation of China (82160643).

## Data availability statement

The data we used is available on the official website of the Peking University Open Research Data. (https://opendata.pku. edu.cn/dataverse/CHADS).

## Ethics statement

The study design was approved by the Ethics Committee of Beijing Hospital. The CLHLS was approved by the Ethics Committee of Peking University (No. IRB00001052-13074). All subjects (or guardians) provided written informed consent before participating in the survey.

## CRediT authorship contribution statement

**Yanrong Wang:** Writing – original draft, Methodology, Investigation, Formal analysis, Data curation, Conceptualization. **Yongbin Zhu:** Writing – review & editing, Formal analysis, Data curation, Conceptualization. **Yueping Wu:** Validation, Supervision, Resources, Project administration. **Liping Shi:** Writing – review & editing, Visualization, Validation. **Yue Yang:** Visualization, Supervision, Software, Resources, Project administration. **Xiaojuan Liu:** Resources, Project administration, Methodology, Funding acquisition. **Jiangping Li:** Resources, Project administration, Methodology, Investigation, Funding acquisition, Data curation, Conceptualization.

## Declaration of competing interest

The authors declare that they have no known competing financial interests or personal relationships that could have appeared to influence the work reported in this paper.

## Figures and Tables

**figs1:**