# Supplementary material for: Association of household chemicals use with cognitive function among Chinese older adults
Source: Heliyon. 2024 Sep 13;10(19):e37765. doi: 10.1016/j.heliyon.2024.e37765 (PMC11466578; doi:10.1016/j.heliyon.2024.e37765)
Supplement: Multimedia component 1 [file mmc1.docx]

**Supplementary material**

**Supplement Table 1.** Measurement of predictive validity of binary regression model.

| **Characteristics** | **Model 1** | **Model 2** | **Model3** |
| --- | --- | --- | --- |
| **AIC** | 6452.164 | 6293.895 | 6187.153 |
| **BIC** | 5880.226 | 6395.371 | 6346.616 |

Note: Model 1 adjusts for age, sex, current residence, and body mass index; Model 2 adjusts for educational attainment and socioeconomic status; Model 3 adjusts for smoking status, alcohol consumption, fruit consumption, and vegetable consumption.

**Supplement Table 2.** Measurement of predictive validity of binary regression model (Stratified Analysis).

| **Subgroup** | **Model 1** | **Model 2** | **Model3** |
| --- | --- | --- | --- |
| **Male** |  |  |  |
| **AIC** | 2286.487 | 2207.927 | 2186.835 |
| **BIC** | 2338.049 | 2291.715 | 2322.185 |
| **Female** |  |  |  |
| **AIC** | 4174.795 | 4100.851 | 4018.925 |
| **BIC** | 4228.029 | 4187.355 | 4158.662 |

Note: Model 1 adjusts for age, current residence, and body mass index; Model 2 adjusts for educational attainment and socioeconomic status; Model 3 adjusts for smoking status, alcohol consumption, fruit consumption, and vegetable consumption.

**Supplement Figure 1.** Using a binary regression model to test the correlation between household chemicals usage and cognitive function scores (Age ≤105). Adjust for age, sex, current residence, body mass index, educational attainment, socioeconomic status, smoking status, alcohol consumption, fruit consumption, and vegetable consumption.

**Supplement Table 3.** A binary regression test was conducted to investigate the correlations between the frequency of household chemicals use and the cognitive function score(Age ≤105).

| **Characteristics** | **Model 1** | | |  | **Model 2** | | |  | **Model 3** | | |
| --- | --- | --- | --- | --- | --- | --- | --- | --- | --- | --- | --- |
|  | **OR** | **95%CI** | ***P*** |  | **OR** | **95%CI** | ***P*** |  | **OR** | **95%CI** | ***P*** |
| **Total score** |  |  |  |  |  |  |  |  |  |  |  |
| **Low- frequency** | Ref. | | |  | Ref. | | |  | Ref. | | |
| **High-frequency** | 2.29 | 1.11-4.73 | 0.025 |  | 2.63 | 1.25-5.55 | 0.011 |  | 2.78 | 1.31-5.91 | 0.008 |

Note: Model 1 adjusts for age, sex, current residence, and body mass index; Model 2 adjusts for educational attainment and socioeconomic status; Model 3 adjusts for smoking status, alcohol consumption, fruit consumption, and vegetable consumption.

**Supplement Table 4.** Linear regression between cognitive function and household chemicals usage in different dimensions (Age ≤105).

| **Characteristics** | **Orientation** | **Registration** | **Calculation** | **Recall** | **Language** |
| --- | --- | --- | --- | --- | --- |
| **insecticide** | -0.21(-0.46, 0.05) | -0.09(-0.18, 0.01) | -0.023(-0.41, -0.04)** | -0.11(-0.22, 0.01) | -0.07(-0.21, 0.08) |
| **Repellents** | -0.01(-0.16, 0.12) | -0.06(-0.11, -0.01)** | -0.01(-0.11, 0.09) | -0.12(-0.18, -0.05)*** | -0.01(-0.09, 0.07) |
| **Anti-caries agent** | -0.09(-0.43, 0.25) | -0.08(-0.21, 0.04) | -0.04(-0.29, 0 .21) | -0.18(-0.33, -0.02)** | -0.15(-0.34, 0.05) |
| **Air freshener** | -0.58(-1.07, -0.09)** | -0.19(-0.36, -0.01)** | -0.30(-0.65, 0.06) | -0.25(-0.47, -0.04)** | -0.21(-0.49, 0.07) |
| **Air purifier** | -0.60(-1.23, 0.02) | -0.24(-0.47, -0.01)** | -0.18(-0.63, 0.28) | -0.28(-0.56, 0.01) | -0.47(-0.83, -0.12)** |
| **Disinfectant** | -0.13(-0.43, 0.17) | -0.08(-0.19, 0.03) | -0.24(-0.45, -0.02)** | -0.10(-0.24, 0.03) | -0.18(-0.35, -0.01)** |
| **Toilet cleaner** | 0.09(-0.08, 0.25) | -0.02(-0.08, 0.04) | -0.04(-0.16, 0.08) | -0.06(-0.13, 0.02) | -0.02(-0.11, 0.08) |
| **Oil remover** | -0.08(-0.25, 0.08) | -0.07(-0.13, -0.01)** | 0.04(-0.08, 0.16) | -0.03(-0.11, 0.04) | -0.06(-0.16, 0.03) |

Note: numerical representation β coefficient (95% CI); **, P<0.05; ***, P<0.01.
